# THE EFFECTIVENESS AND COSTS OF INTENSIVE STROKE REHABILITATION AND IMPROVEMENTS IN PATIENT PATHWAY IN FINLAND: A RETROSPECTIVE BENCHMARKING CONTROLLED TRIAL

**DOI:** 10.2340/jrm.v56.34944

**Published:** 2024-11-13

**Authors:** Niko KORPI, Marja MIKKELSSON, Unto HÄKKINEN, Antti MALMIVAARA

**Affiliations:** 1 Doctoral Programme in Clinical Research University of Helsinki, and Head Physician, Orimattila Health Center, Wellbeing Services County of Päijät-Häme; 2 University of Helsinki, Department of Rehabilitation, Wellbeing Services County of Päijät-Häme Lahti; 3 Finnish Institute for Health and Welfare, Helsinki; 4 Assessment of the Health and Social Service System, National Institute for Health and Welfare, Helsinki; 5 Orton Orthopedic Hospital, Helsinki, Finland

**Keywords:** stroke, rehabilitation, rehabilitation centre, interprofessional, rehabilitation pathway, PERFECT, effectiveness, costs

## Abstract

**Objective:**

To assess the effectiveness and costs of intensive stroke rehabilitation and improvements in patient pathway in the city of Lahti and in Päijät-Häme region compared with other parts in Finland.

**Design:**

Retrospective benchmarking controlled trial.

**Patients:**

Three cohorts of Finnish community-dwelling patients (*n* = 94,749, *n* = 4,184, and *n* = 105,458) with ischaemic stroke between 2001 and 2019.

**Methods:**

This study is based on the PERFECT 2001–2019 database of ischaemic stroke patients. PERFECT indicators describe how the stroke patients recover. The difference-in-difference method was used in the main analysis.

**Results:**

Improved stroke rehabilitation in Lahti increased the share of patients discharged home (*p* = 0.005) and decreased the length of first institutional episode (–4 days, *p* = 0.006), the share of patients institutionalized (–5.1%, *p* = 0.001), and the costs of first institutional episode (€–2,085, *p* < 0.001) compared with the rest of Finland. Discharges to home increased 6.6 percentage points (*p* = 0.021) in Lahti compared with rest of Päijät-Häme. After 2013, the costs of first institutional episode per patient in Päijät-Häme decreased significantly compared with the rest of Finland (*p* < 0.001).

**Conclusion:**

Investments in intensive stroke rehabilitation and patient pathway seem to provide both faster and better return to home for patients and reduced costs for the healthcare system.

## LAY ABSTRACT

This retrospective benchmarking controlled trial aimed to assess the effectiveness and costs of intensive stroke rehabilitation and improvements in patient pathway in the city of Lahti in 2006 and in Päijät-Häme region, Finland, in 2013. Data were gathered from the national PERFECT database. The data of stroke patients from Lahti were compared with other municipalities of Päijät-Häme and with the rest of Finland. Investments in intensive stroke rehabilitation and patient pathway seem to provide both faster and better return to home for patients and reduced costs for the healthcare system.

Acute stroke care, including interprofessional rehabilitation in specialized units, can reduce disability and mortality in ischaemic stroke (1–3). Although improved acute stroke care has improved the prognosis of stroke (4–6), the role of interprofessional rehabilitation in specialized stroke rehabilitation centres or stroke units is still important (7–9). Home-based rehabilitation services can be designed to enable patients to return home earlier and support self-rehabilitation efforts (10, 11).

In Finland, after the patient’s condition has stabilized in the acute phase, the patient is transferred from the university hospital or from the central hospital to an interprofessional rehabilitation unit (12). Although the effectiveness of interprofessional rehabilitation was already shown over two decades ago (4–6), the availability of acute stroke centres and specialized stroke rehabilitation centres has varied between Finnish municipalities and hospital districts (13–15).

As many regions in Finland are sparsely populated, rehabilitation wards have been acute medical wards incorporated as part of health centres and many of them have had only a few stroke care episodes each year (13). Only 15% of Finnish ischaemic stroke patients receive appropriate interprofessional rehabilitation (14, 15). The lack of proper therapies was evident (14, 15). The same problem exists in Finland’s neighbouring country Estonia, which has good acute care in overall but rehabilitation services seem to be lacking (16). Internationally the resource situation is somewhat unclear. Most countries do not monitor their provided rehabilitation services extensively and comparisons are difficult to make. (17)

Our aim was to assess the effectiveness and costs of intensive stroke rehabilitation and improvements in patient pathway in the city of Lahti and in Päijät-Häme region compared with other parts in Finland.

## METHODS

This study is a retrospective benchmarking controlled trial (18) of Finnish community-dwelling patients. The study is based on the PERFECT (Performance, Effectiveness and Cost of Treatment episodes) 2001–2019 database of stroke patients, which is based on linkable, patient-level data on incident stroke patients (19).

The need for ischaemic stroke pathway improvements in Lahti was noticed during the pre-interventional period from 2001 to 2005. The improvements were made in 2 phases. In the first phase the Päijät-Häme’s regional capital city Lahti started to provide additional support for rehabilitation of ischaemic stroke patients by forming a neurological rehabilitation ward in 2006 and a home-based rehabilitation team to support discharge to home in 2007. The team provides rehabilitation services to community-dwelling stroke survivors, including varied therapies, assistive devices, and home conversion work. At the same time, stroke rehabilitation in other municipalities of the hospital district was performed in health centre wards. The following year, 2008, the board of Päijät-Häme joint municipal authority started a project to improve interprofessional stroke rehabilitation. This project led to reinforcement of the regional interprofessional stroke rehabilitation and establishment of a regional stroke rehabilitation unit.

In the second phase the stroke rehabilitation centre of Lahti took responsibility for regional stroke rehabilitation in Päijät-Häme in 2013. The stroke rehabilitation unit included a specialist in neurology and physiatry, physiotherapists, occupational therapists, speech therapists, and neuropsychologists. The nursing team was trained in rehabilitative working methods, which have previously been shown to be effective (20). The home-based rehabilitation team gradually expanded to cover the whole region until complete regional coverage was achieved in 2017. Here, we analysed the effects of the 2 interventions by describing time trends in outcome indicators in intervention regions and control groups and using the difference-in-difference (DID) method similarly to a recent study on hip fracture (21).

In Finland, the PERFECT project contains databases of patients that monitors treatment episodes in specialized healthcare. It was started by the National Research and Development Centre for Welfare and Health together with Finnish University Hospitals. The project created performance indicators and models for monitoring and assessment of healthcare content, quality, and costs. Stroke is one of the diseases monitored by the PERFECT project (19). The methodology has been extended to international comparisons (22). It has already been shown by Meretoja et al. (23) that stroke outcomes in Finland have been improved. Effectiveness of a clinical pathway in real-life circumstances is often best assessed by study design, a benchmarking controlled trial utilizing register data, in this case the PERFECT database (18, 24, 25). Population-wide register data such as the PERFECT projects enable the comparison of regional outcomes of stroke care (19, 21).

The construction of the data was based on a common protocol using routinely collected national registers and statistics on hospital discharges, the use of prescribed medication, and causes of death (26). For each patient, all continuous hospital treatment (from the first hospital episode) starting from the first stroke (cerebral infarction [ICD-10 code I63], intracerebral haemorrhage [ICD-10 code I61], subarachnoid haemorrhage [ICD-10 code I60], or an ill-defined stroke [ICD-10 code I64]) admission (index admission) in every year was constructed by combining all consecutive hospital stays for each patient. The consecutive hospital stays did not need to be in the same hospital; hospital transfers were considered when constructing the first hospital episode. In the case where a patient had different stroke subtypes or ill-defined stroke diagnoses during the first hospital episode, the most “severe” diagnosis was chosen to characterize the patient’s condition. For this purpose, the following hierarchy of stroke subtypes was applied: subarachnoid haemorrhage, intracerebral haemorrhage, cerebral infarction, and ill-defined stroke. The same patient could have been included several times in the cohort, if the end of the earlier stroke hospital admission (all stroke types) and a new stroke admission (all stroke types) was longer than 365 days. However, the effect of this possibility had been taken into consideration by including the previous stroke as a comorbidity variable that was used in the risk adjustment (Appendix S1).

To increase data comparability, we included only cerebral infarction (an ischaemic stroke) based on the above-mentioned hierarchy using 2 exclusions. First, we excluded all patients with a stroke admission (a hospital discharge record with a stroke diagnosis as the main diagnosis) during the previous 365 days before the index admission. Second, we excluded foreign nationals and patients with an incomplete personal identity number, because they were not included in the database and their comorbidities were unknown. Third, we excluded patients who were in long-term institutional care before the index admission. The validation of stroke diagnoses has been published previously (27).

We applied the following performance measures, similar to those in the recent PERFECT hip fracture study (21) and in a Nordic comparison of stroke patients (22): length of first acute-care admission, length of the first institutional episode (including, e.g., acute care and rehabilitation), proportion of patients discharged home within 90 days and still alive at the end of the period, share of patients institutionalized (90 days), number of inpatient days in 1 year, 90-day mortality, 1-year mortality, cost of first institutional episode (€/patient), and the 1-year cost of inpatient care (€/patient). The use of inpatient care during the first institutional episode and 1 year were converted into costs at the 2020 price level, using the information on diagnosis-related groups (DRGs) from the acute hospital care and the type of provider (such as health centre or nursing home) from other institutions.

The conversion had been done in the following way. First, we classified all acute care admissions from the study period using Finnish version of the NordDRG grouper (28) from the year 2020. Then we calculated the cost per day for each of the DRG group of the patients who had admission-level data available in the Finnish discharge register. We translated acute hospital care to monetary terms using annual median cost per day including all acute admissions. In psychiatry we used median cost per day in the speciality as DRG was not usable. In long-term care we used Finnish standard cost estimates per day for different types of non-acute and non-psychiatric care (29).

During the study period, the Finnish registers included data only on hospital care, including health centres and nursing homes. The data included most of the stroke rehabilitation efforts, as they were mostly undertaken in the inpatient wards in Finland. In addition, it should be noted that inpatient care accounts for most of the 1-year costs of stroke patients. From the data of a recent study (22) using a rather similar cohort of ischaemic stroke patients, it can be estimated that about 90% of all 1-year costs were accounted to inpatient care in the Helsinki area in 2014. Since then, the use of outpatient services has somewhat increased in the care of stroke patients. Nonetheless there is no information as to whether the increase has been higher in the study groups than in the control groups used in this study.

The DID method is currently a widely used quasi-experimental statistical technique in econometrics and quantitative research in social sciences. It attempts to mimic an experimental research design using observational study data, by studying the differential effect of a treatment on a “treatment group” versus a “control group”. Here we followed a similar approach to that recently used in analysis of interventions on hip fracture patients (21).

The DID method uses trends in the regions that participated in the intervention and in the region that did not before and after the intervention and estimates the changes made by the intervention. In this study the DID method is applied to individual-level data using information concerning the patients’ home region. In DID analysis, we used the same patient covariates as control variables (Appendix SI). We also report the marginal effects of outcome variables (based on the appropriate regression models and adjusted by the control variables and year indicators) at the baseline (i.e., time before the interventions). Because applying nonlinear models within a DID framework is challenging and leads to inconsistent estimates of the effect (31) our DID analyses were based on the ordinary least-squares (OLS) models. For sensitivity analysis, we combined DID with propensity matching and used bootstrapped standard errors (32) using the default Epanechnikov Kernel function with a bandwidth of 0.06. Bootstrapping was made using 5,000 replicates.

To estimate any causal effect, the DID method should fulfil several assumptions, of which the common trend assumption is the most important in our case. The common trend assumption requires that the (case-mix adjusted) trends of outcomes should have similar shapes between treatment and control groups, also after the intervention in the imaginary case of the absence of intervention. As this assumption is based on non-observable counterfactuals, there is no formal test. However, we performed a simple investigation of the assumption by estimating an OLS model explaining the outcome indicators by intervention area variable with the covariates and including annual indicators or a time trend (linear, logarithmic, and quadratic trends) and their interactions with the reform variables, using the pre-interventional data before the reforms. Annual year variable is a dichotomic year indicator (e.g., for year 2005 value = 1 if patient belongs to cohort 2005, otherwise value = 0) and it was tested to ascertain whether their interactions were significant with the intervention variables. A similar test has been used in earlier studies (21, 30).

The implementation of the interventions took time, and their effects were not apparent immediately after implementation date (e.g., the reform in Lahti was implemented gradually during 2006). Thus, we excluded the first year after the reform from DID analysis (e.g., 2006 in analysis of the Lahti intervention and 2013 in analysis of the Päijät-Häme intervention). Lahti was compared with the rest of Päijät-Häme and the rest of Finland in 2007–2012 by using pre-intervention patient cohorts in 2001–2005. Päijät-Häme was compared with the rest of Finland in 2014–2017 by using pre-intervention patient cohorts of the years 2007–2012.

The results were illustrated by figures that describe time trends. The figures were based on marginal effects using a repeated analysis technique for the 365-day cohorts. We performed a repeated (by 30 days) regression analysis so that each monthly cohort consisted of patients whose episode began on that date or during the next 365 days. We performed 220 repeated analyses, the first of which included patients with an index day between 1 January 2001 and the following 365 days; the second between 31 January 2001 and the following 365 days; and the last between 28 December 2018 and the following 365 days.

Trends in figures were estimated using appropriate regression models, namely logistic regression for dichotomous responses (e.g., emplacement and mortality variables), negative binomial modelling for discrete count variables (e.g., length of stay), and generalized linear modelling for continuous variables (e.g., costs were modelled using a gamma distribution with log link). The marginal effects were measured using dummy variables describing the regions. In addition, various patient covariates (such as age, sex, and 16 comorbidities) were controlled in all models (Appendix S1). In figures, we reported only time-trend intervals of the performance variables where the trends differed between the groups.

The whole PERFECT cohort used in this study and the figures consisted of 165,114 patients. The DID analysis was made using 3 patient cohorts. First, we compared the patient cohorts of Lahti with the rest of Finland (Table I) in the years 2001–2005 and 2007–2012 (*n* = 94,749). Second, we compared patient cohorts between Lahti and other municipalities of Päijät-Häme (Table II) in the years 2001–2005 and 2007–2012 (*n* = 4,184). Finally, we analysed whether starting the regional rehabilitation ward in 2013 influenced the estimates of Päijät-Häme (including Lahti) compared with the rest of Finland (Table III) using patient cohorts 2007–2012 and 2014–2019 (*n* = 105,458).

**Table I:** Comparison of effectiveness and costs between Lahti (2007–2012) and rest of Finland

Factor	Marginal estimates before the intervention 2001–2005		DID analysis				
			Analysis of parallel trend assumption 2001–2005		DID estimates (2001–2005, 2007–2012)		
	Lahti^a^	Rest of Finland	Annual year variables	Trend^b^	DID		DID (pm and bs)^c^
Number of patients	778	41,596	42,374		94,749		
Length of first acute care admission, days	11.7 (0.184)	12.2	***	**	-2.5 (0.000***)		-2.6 (0.000***)
Length of first institutional episode, days	30.3 (0.001**)	27.0	ns	ns	-4.4 (0.001**)		-4.0 (0.006**)
Share of patients discharged home within 90 days (%)	66.8 (0.000***)	72.7	ns	ns	6.8 (0.000***)		6.1 (0.005**)
Share of patients institutionalized (90 days) (%)	16.8 (0.000***)	11.5	ns	ns	-5.4 (0.000***)		-5.1 (0.001**)
Number of inpatient days, 1 year	71.6 (0.030*)	65.0	ns	ns	-8.5 (0.055)		-7.1 (0.131)
90-day mortality (%)	15.9 (0.270)	14.5	ns	ns	-1.4 (0.380)		-0.9 (0.575)
One-year mortality (%)	23.6 (0.113)	21.3	ns	ns	-2.1 (0.229)		-1.5 (0.433)
Cost of first institutional episode (€/patient)	12,040 (0.005**)	10,774	ns	ns	-2,206 (0.000***)		-2,085 (0.000***)
One-year cost of inpatient care (€/patient)	26,332 (0.039*)	23,750	ns	ns	-3,421 (0.020*)		-3,050 (0.056)

a In parentheses *p*-values of coefficients of a dichotomic variable describing whether a patient is living in Lahti.

b Indicates the highest significance among linear, logarithmic, or quadratic trend.

c With propensity matching and bootstrapped standard errors (32).

ns: not significant.

* *p* < 0.05.

** *p* < 0.01.

*** *p* < 0.00.

**Table II:** Comparison of effectiveness and costs between Lahti (2007–2012) and other municipalities in Päijät-Häme (Other Päijät-Häme)

Factor	Marginal estimates before the intervention (2001–2005)		DID analysis				
			Analysis of parallel trend assumption (2001–2005)		DID estimates (2001–2005, 2007–2012)		
	Lahti^a^	Other Päijät-Häme	Annual year variables	Trend^b^	DID	DID (pm and bs)^c^	
Number of patients	778	949	1,727		4,184		
Length of first acute care admission, days	11.7 (0.000***)	8.5	***	**	-2.8 (0.000***)	-2.8 (0.000***)	
Length of first institutional episode, days	30.2 (0.037*)	27.2	ns	ns	-1.3 (0.463)	-1.4 (0.474)	
Share of patients discharged home within 90 days (%)	67.6 (0.006**)	73.5	ns	ns	6.4 (0.014*)	6.6 (0.021*)	
Share of patients institutionalized (90 days) (%)	16.7 (0.008**)	12.2	ns	ns	-4.0 (0.047*)	-4.1 (0.055)	
Number of inpatient days, 1 year	70.1 (0.508)	67.3	ns	ns	2.4 (0.687)	2.0 (0.750)	
90-day mortality (%)	15.2 (0.218)	13.2	ns	ns	-2.6 (0.216)	-2.7 (0.230)	
One-year mortality (%)	22.2 (0.190)	19.8	ns	ns	-3.3 (0.159)	-3.2 (0.211)	
Cost of first institutional episode (€/patient)	12,099 (0.009**)	10,554	ns	ns	-872 (0.202)	-872 (0.237)	
One-year cost of inpatient care (€/patient)	26,057 (0.374)	24,504	ns	ns	584 (0.769)	600 (0.782)	

a In parentheses *p*-values of coefficients of a dichotomic variable describing whether a patient is living in Lahti.

b Indicates the highest significance among linear, logarithmic, or quadratic trend.

c With propensity matching and bootstrapped standard errors (32).

ns: not significant.

* *p* < 0.05.

** *p* < 0.01.

*** *p* < 0.001.

**Table III:** Comparison of effectiveness and costs between Päijät-Häme (Lahti included) (2007–2012 and 2014–2019) and rest of Finland

Factor	Marginal estimates before the intervention (2007–2012)		DID analysis				
			Analysis of parallel trend assumption (2007–2012)		DID estimates (2007–2012, 2014–2019)		
	Päijät-Häme^a^	Rest of Finland	Annual year variables	Trend^b^	DID	DID (pm and bs)^c^	
Number of patients	2457	49918	52375		105458	
Length of first acute care admission, days	7.1 (0.000***)	10.6	ns	ns	-2.4 (0.000***)	-2.5 (0.000***)	
Length of first institutional episode. days	22.9 (0.000***)	24.7	*	*	-1.8 (0.024*)	-1.4 (0.074)	
Share of patients discharged home within 90 days (%)	76.8	76.2	ns	ns	2.6	1.5	
	(0.453)				(0.020*)	(0.184)	
Share of patients institutionalized (90 days) (%)	9.5 (0.538)	9.9	ns	ns	-1.2 (0.147)	-1.0 (0.207)	
Number of inpatient days, 1 year	53.7 (0.000***)	58.9	***	***	-3.4 (0.185)	-1.5 (0.557)	
90-day mortality (%)	12.9 (0.428)	12.4	*	**	-1.4 (0.113)	-0.6 (0.498)	
One-year mortality (%)	18.8 (0.670)	18.4	*	*	-1.1 (0.284)	0.0 (0.995)	
Cost of first institutional episode (€/patient)	9,154 (0.000***)	10,434	ns	ns	-1,799 (0.000***)	-1,714 (0.000***)	
One-year cost of inpatient care (€/patient)	19,485 (0.000***)	21,688	***	***	-3,051 (0.000***)	-2,591 (0.001**)	

a In parenthesies *p*-values of coefficients of a dichotomic variable describing whether a patient is living at Päijät-Häme.

b Indicates the highest significance among linear, logarithmic, or quadratic trend.

c With propensity matching and boot-strapped standard errors (32).

ns: not significant.

* *p* < 0.05.

** *p* < 0.01.

*** *p* < 0.001.

In each table the second and third columns describe the marginal effects of dependent variables in the intervention and control group before the intervention. The fourth and fifth columns describe the analysis of parallel trend assumption in both groups before intervention. The sixth and seventh columns describe DID estimates using data for all years as indicated in the tables and text. We used the significance levels of 5%, 1%, and 0.1% (described with asterisks in the tables).

The study was approved by the respective patient registry holder of Päijät-Häme Central Hospital. Ethical committee approval was not required, as patients were not contacted and were not identifiable by the authors.

## RESULTS

### Comparison of Lahti and rest of Finland

First, we compared the patient cohorts of Lahti with the rest of Finland in the years 2001–2005 and 2007–2012. Before the intervention, the proportion of patients discharged home was about 6 percentage points lower (*p* < 0.001), the proportion of patients institutionalized was 5 percentage points higher (*p* < 0.001), the length of first institutional episode was 3 days longer (*p* = 0.001), and 1-year inpatient days were 7 days higher in Lahti compared with the rest of Finland (*p* = 0.03). There were no statistically significant differences in the 90-day mortality or 1-year mortality. The costs of the first institutional episode and the 1-year costs of inpatient care were about €1,270 (*p* = 0.005) and €2,580 (*p* = 0.039) higher in Lahti compared with the rest of Finland, respectively (Table I and Figs 2–7).

**Fig. 1:**
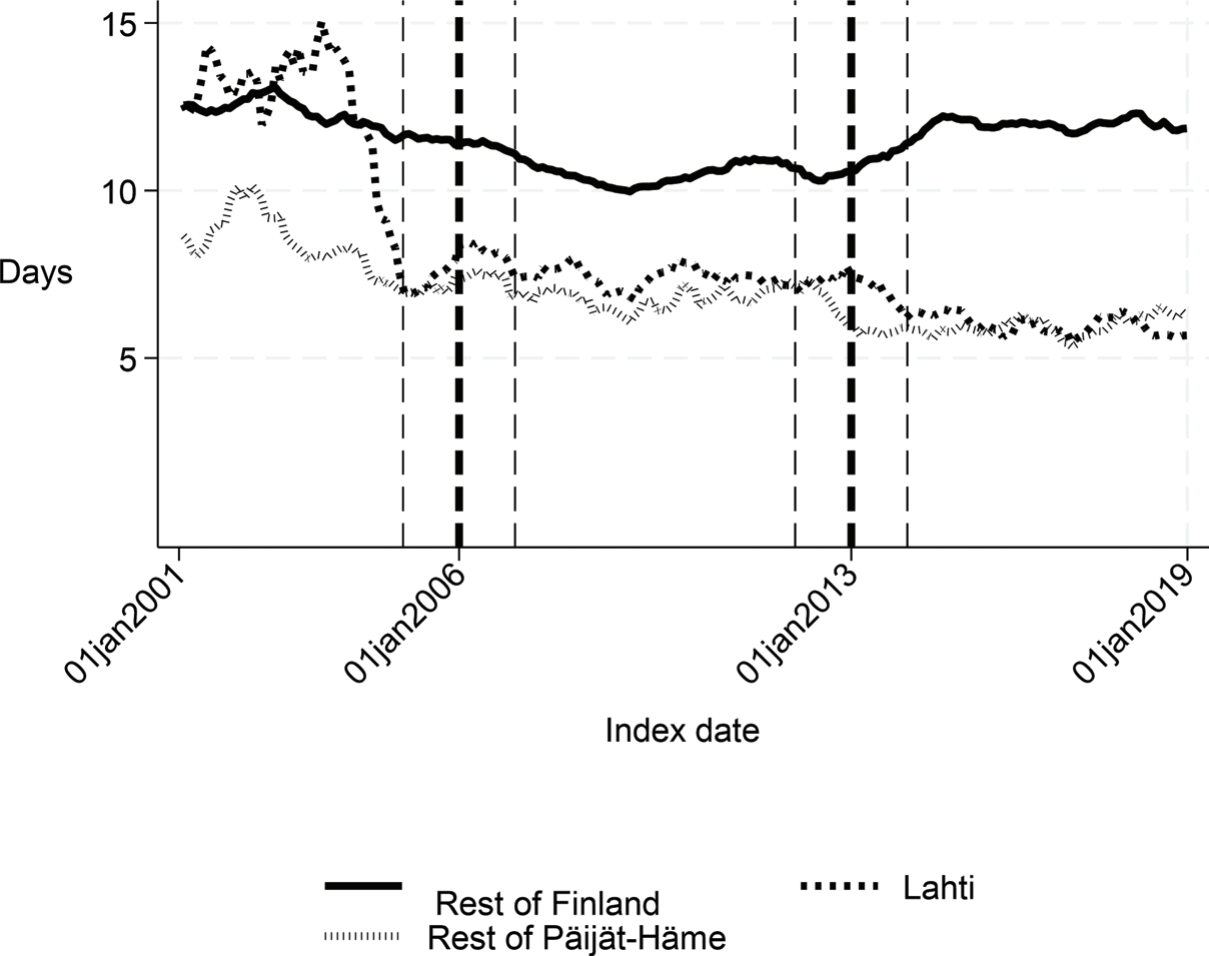
Length of first acute care admission in Lahti, in rest of Päijät-Häme, and in rest of Finland 2001–2019 (days).

The length of the first institutional episode decreased by 4 days (*p* = 0.006) and the share of patients institutionalized within 90 days decreased by 5 percentage points (*p* = 0.001) in favour of Lahti. The DID estimate for the length of the first acute care admission was reduced by almost 3 days (*p* < 0.001) in Lahti compared with the rest of Finland, although Fig. 1 and the statistical evaluation strongly suggest a violation of the common trend assumption. The cost of first institutional episode decreased by over €2,000 per patient in favour of Lahti (*p* < 0.001), which is about 20% smaller than the average cost per patient in the whole sample (Appendix S1). Although the 1-year cost of inpatient care decreased by over €3,000, the change was not statistically significant (*p* = 0.056) in the sensitivity analysis. There were no statistically significant changes in other performance measures.

### Comparison of Lahti and the rest of Päijät-Häme

**Fig. 2:**
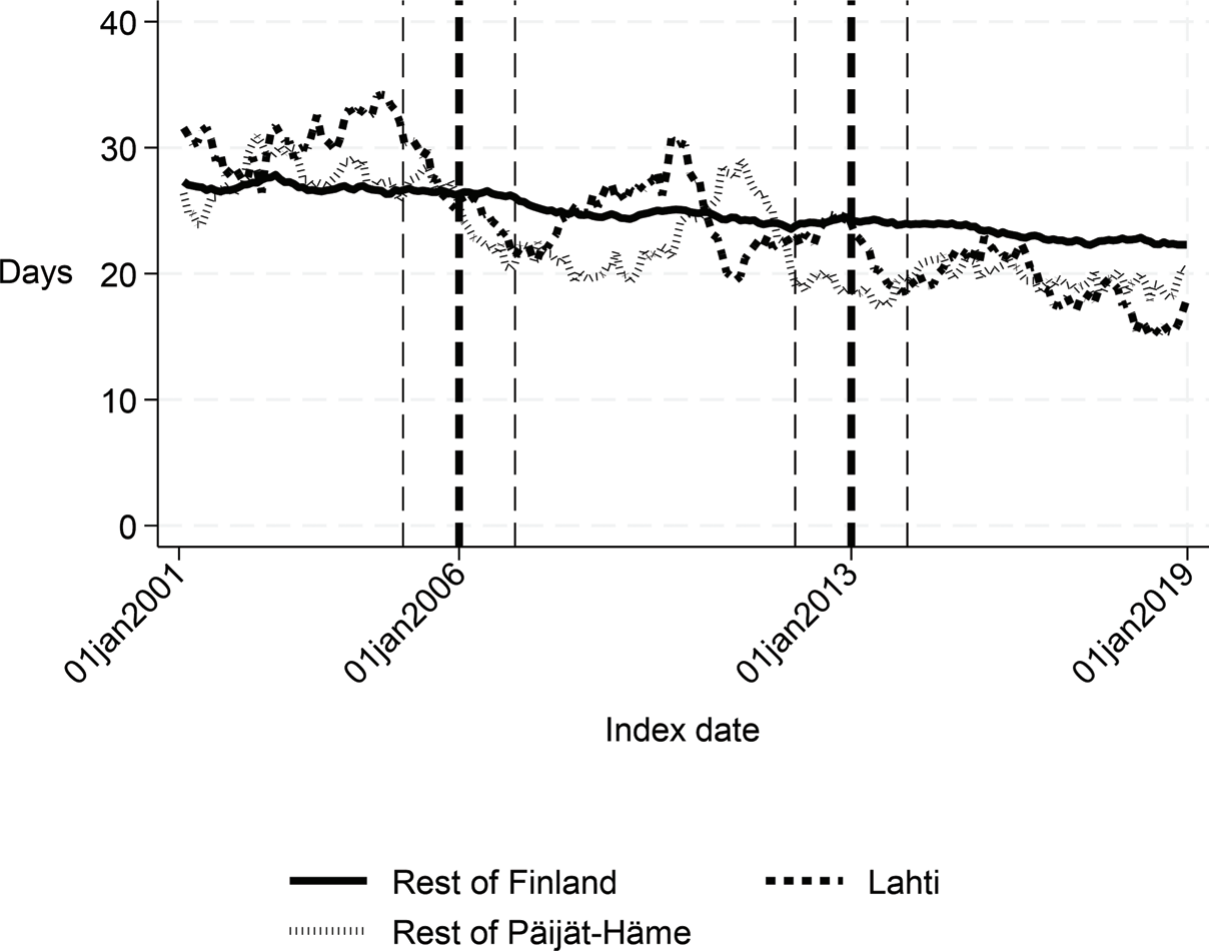
Length of first institutional episode in Lahti, in rest of Päijät-Häme, and in rest of Finland 2001–2019 (days).

**Fig. 3:**
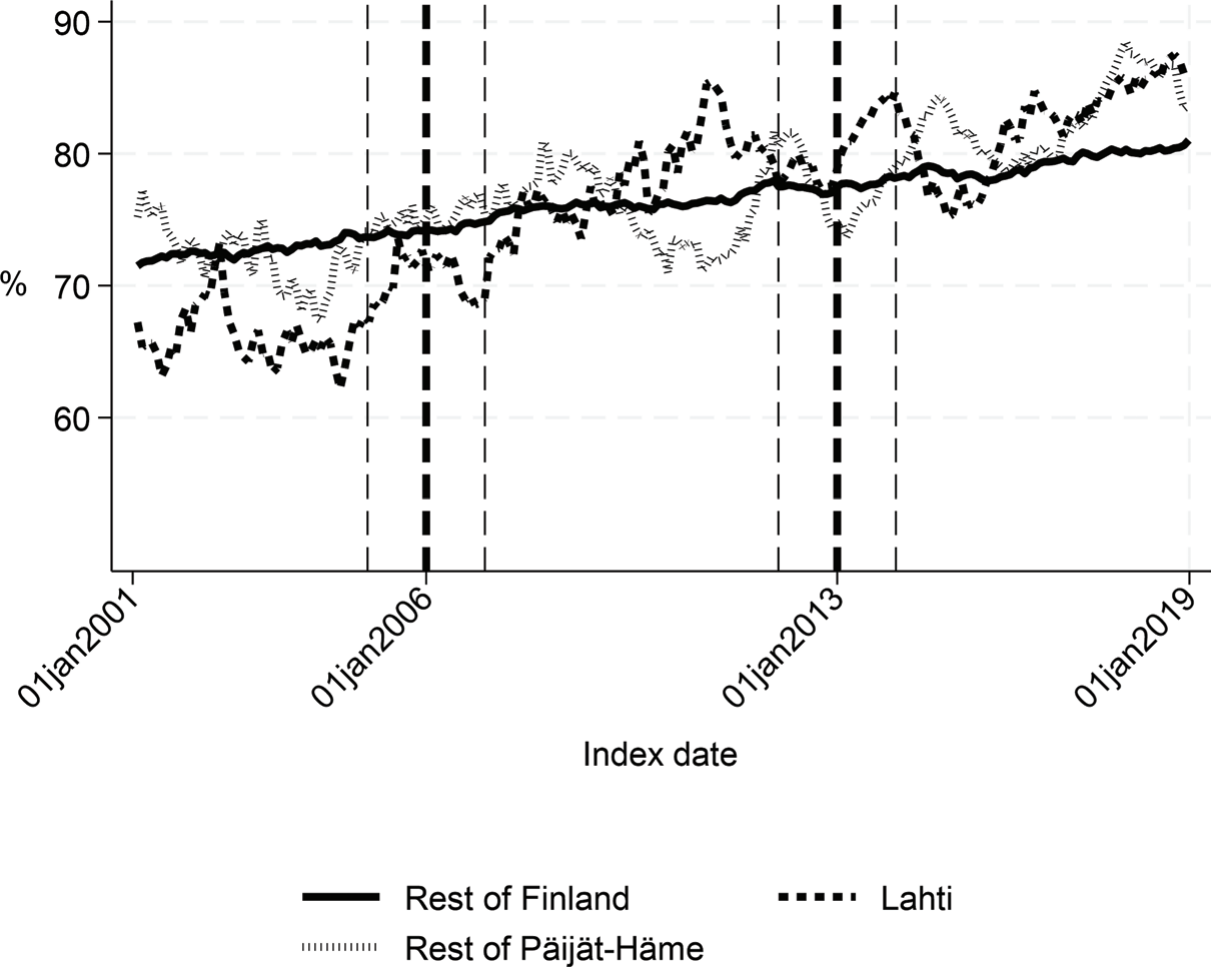
Share of patients discharged home within 90 days in Lahti, in rest of Päijät-Häme, and in rest of Finland 2001-2019 (%).

**Fig. 4:**
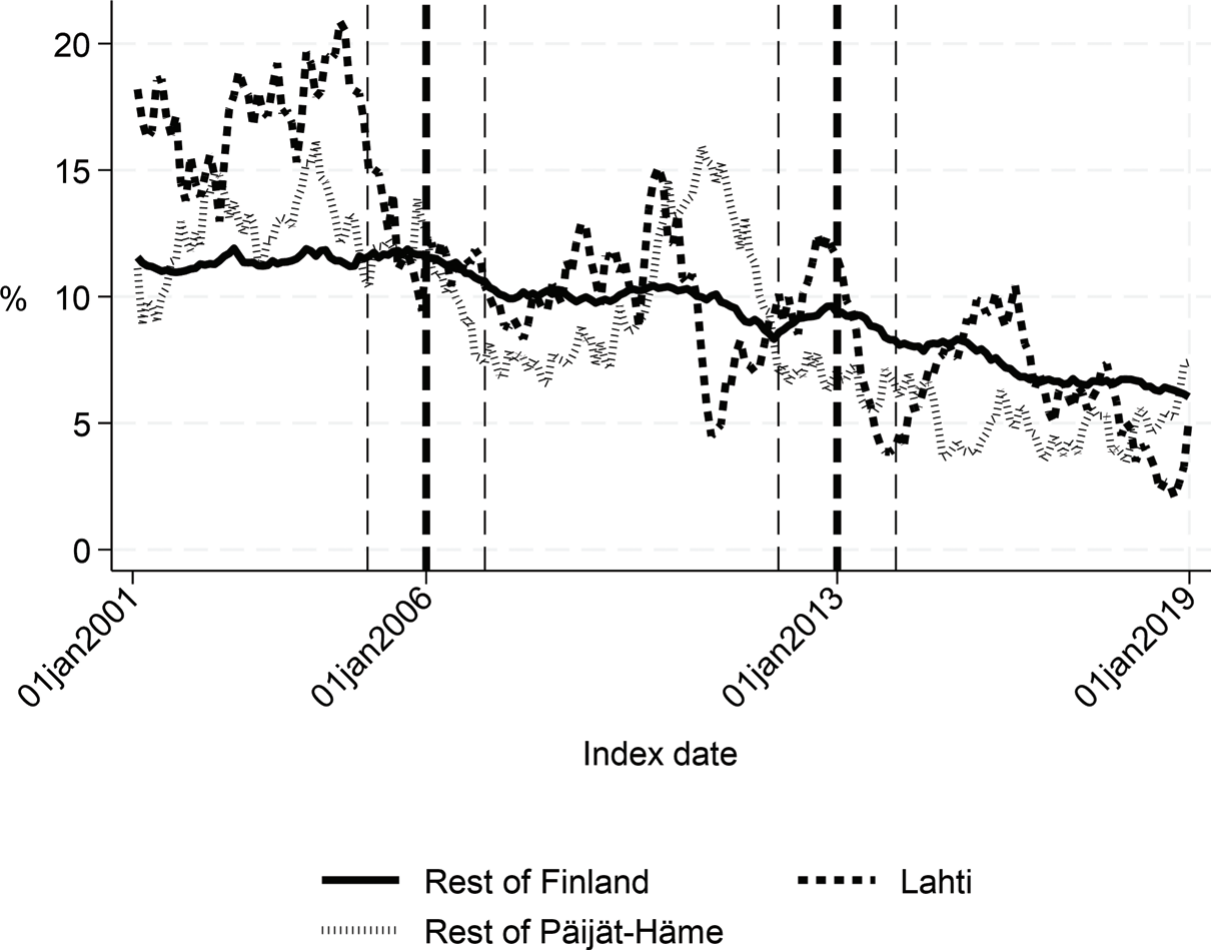
Share of patients institutionalized within 90 days in Lahti, in rest of Päijät-Häme, and in rest of Finland 2001–2019 (%).

**Fig. 5:**
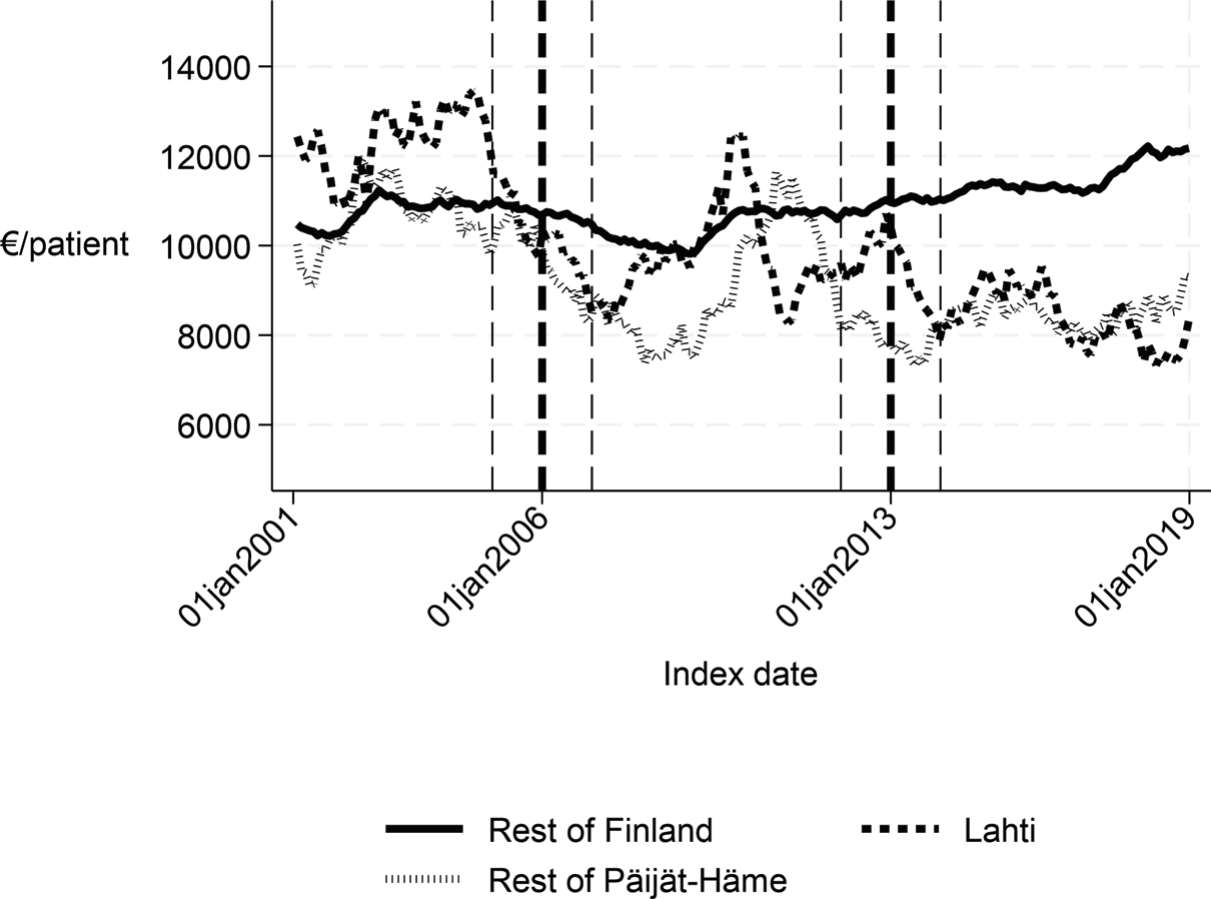
Cost of the first institutional episode in Lahti, in rest of Päijät-Häme, and in rest of Finland 2001–2019 (€/patient).

Before the intervention in 2006, the length of first acute care and the first institutional episode were about 3 days longer in Lahti compared with the rest of Päijät-Häme (*p* < 0.001 and *p* = 0.037 respectively). In addition, the proportion of patients discharged home was lower in Lahti (*p* = 0.006). The proportion of patients institutionalized within 90 days (*p* = 0.008) and costs of first institutional episode (*p* = 0.009) were higher in Lahti (Figs 1–5 and Table II) than in the rest of Päijät-Häme.

Long-term trends revealed changes in favour of Lahti in length of first acute care admission (Fig. 1), length of first institutional episode (Fig. 2), proportion of patients discharged home within 90 days (Fig. 3), and proportion of patients institutionalized within 90 days (Fig. 4). According to DID estimates, the proportion of patients discharged home within 90 days increased 6.6 percentage points in favour of Lahti (*p* = 0.021) (Table II). The proportion of patients institutionalized decreased in Lahti compared with the rest of Päijät-Häme, but this was not significant in the sensitivity analysis. Again, the DID estimate for the length of first acute care revealed a decrease of about 3 days (*p* < 0.001) in Lahti compared with the rest of Päijät-Häme. In Fig. 1 the statistical evaluation strongly suggests a violation of the common trend assumption.

### Comparison of Päijät-Häme and the rest of Finland

Before the intervention in 2013, the length of the first acute care admission, the first institutional episode, number of inpatient days during the first year, and both cost indicators were significantly lower in Päijät-Häme compared with the rest of the country (*p* < 0.001). After the intervention, length of first acute-care admission and cost of first institutional episode decreased significantly compared with the rest of Finland (*p* < 0.001). The decreased cost by about 1,800€ per patient is about 16% of the average cost per patient in the whole sample. Although the 1-year cost of inpatient care (*p* = 0.001) and number of inpatient days decreased, again the analysis suggested a violation of the common trend assumption.

**Fig. 6:**
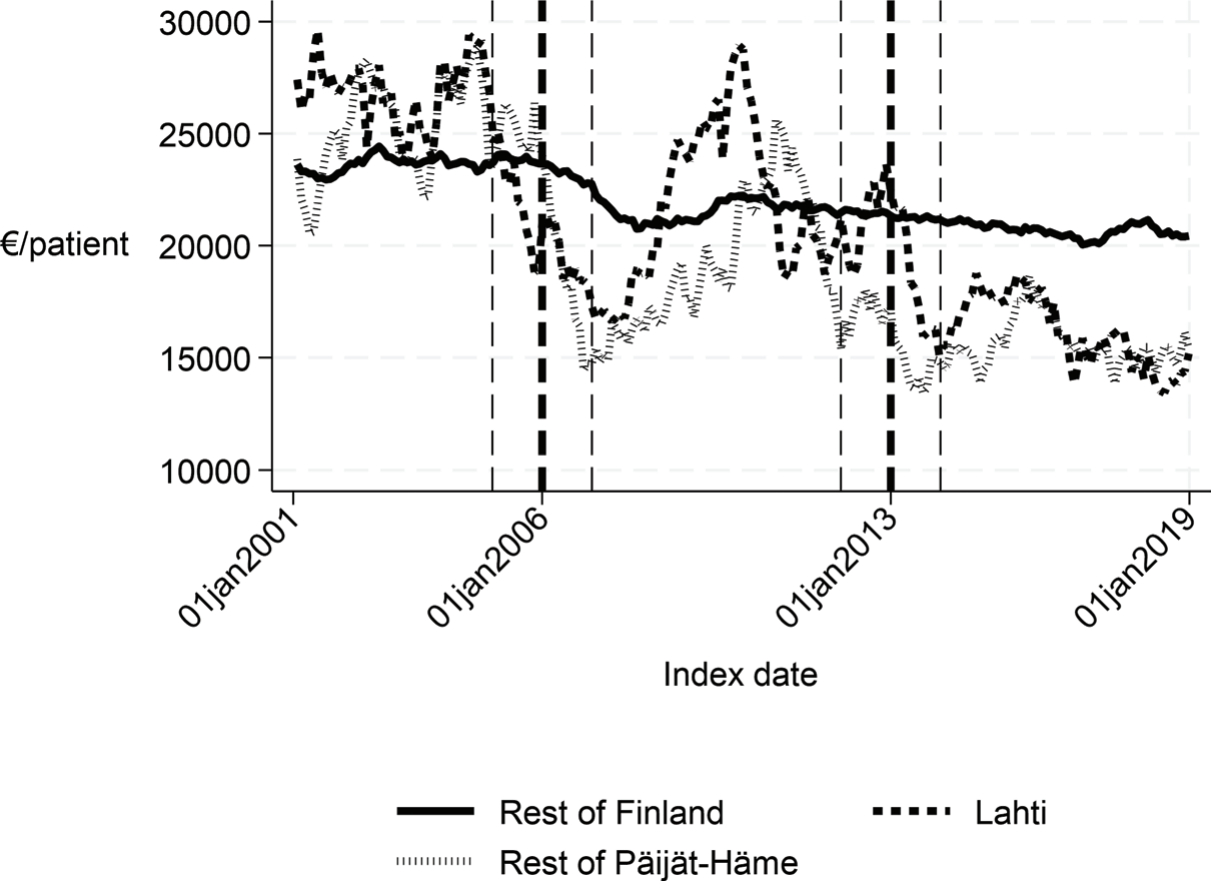
One-year cost of inpatient care in Lahti, rest of Päijät-Häme, and rest of Finland 2001–2019 (€/patient).

Long-term trends of indicators suggest that compared with the rest of Finland, Lahti and the rest of Päijät-Häme have shorter first acute-care admission (in Lahti after 2006 intervention, see Fig. 1), have improved in the length of first institutional episode (see Fig. 2), have achieved and exceed the level of the rest of Finland in proportion of patients discharged home within 90 days (see Fig. 3), and achieved the level of the rest of Finland in proportion of patients institutionalized within 90 days (see Fig. 4). There was a decreasing trend of costs in Päijät-Häme both in first institutionalized episode (Fig. 5) and in one-year costs of inpatient care compared with the rest of Finland (Fig. 6).

**Fig. 7:**
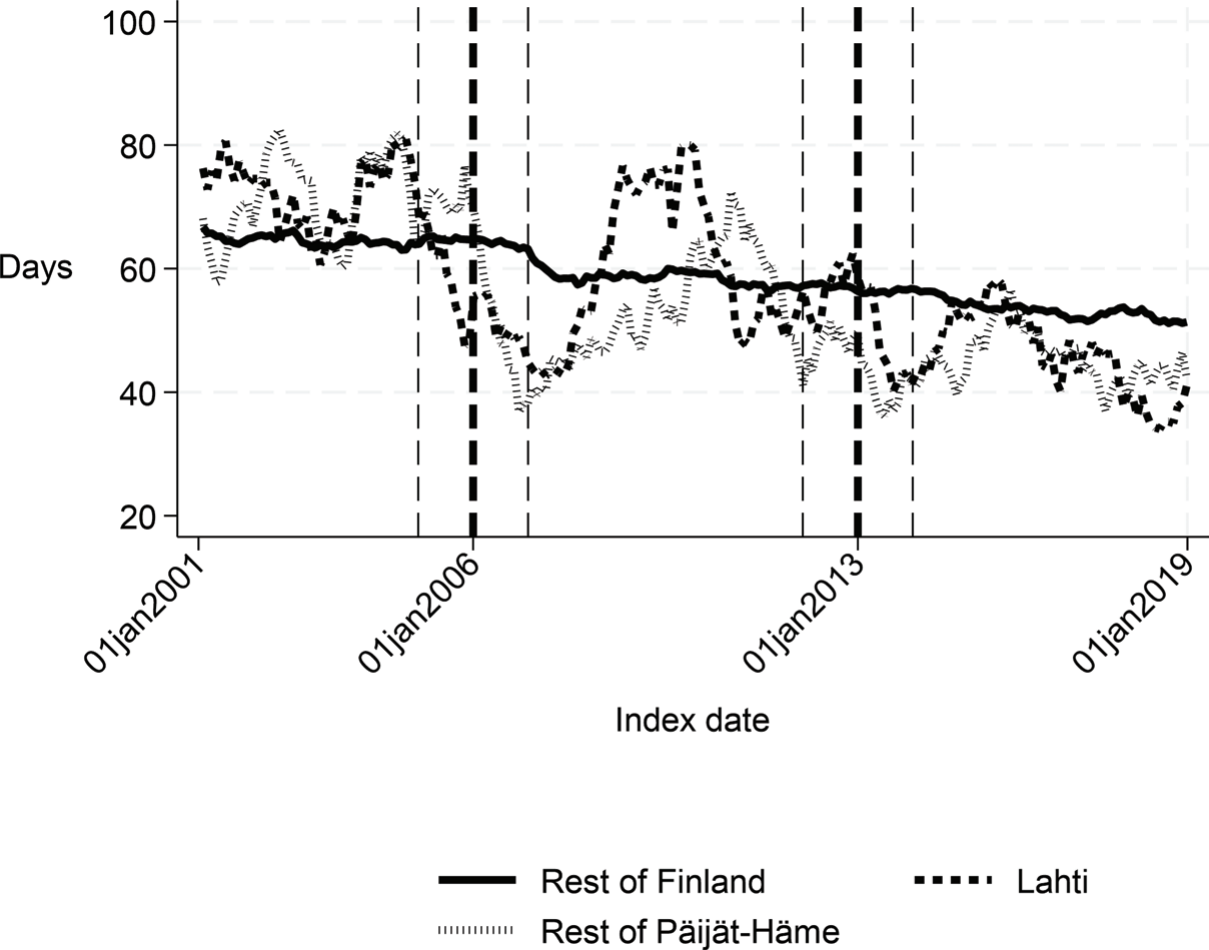
Number of inpatient days within 365 days: Lahti, rest of Päijät-Häme, and rest of Finland 2001–2019.

## DISCUSSION

This study indicated that Lahti, which had improved practices for stroke rehabilitation, reduced the length of first acute-care admission and the first institutionalized episode permanently. In addition, Lahti had fewer patients institutionalized in 90 days compared with the rest of Finland. After the regional intervention in 2013, the days of the first admission care decreased compared with the rest of Finland, and the cost of the first institutional episode decreased.

The performance of stroke care in Lahti estimated by PERFECT indicators before 2006 was weaker than in the rest of Finland and in other municipalities of Päijät-Häme. These indicators indirectly measure the performance at different levels of the care pathway. The 2006 intervention in Lahti aimed to shorten acute-care admission and to improve the quality of rehabilitation at the primary care hospital. The Päijät-Häme 2013 intervention aimed to achieve equal possibilities to receive interprofessional rehabilitation and home-based rehabilitation for all inhabitants of the Päijät-Häme region.

One important aim of rehabilitation is to improve functional ability such that patients can return home, and subsequent institutionalized care is not needed. The requirement for institutional care, especially in Finland, indicates disability that precludes independent living at home. The long-term results showed that Lahti reduced the length of first acute-care admission and the first institutionalized episode (including acute care and rehabilitation) permanently (follow-up of 13 years). Improved integration between specialized care and primary care and more efficient rehabilitation may explain these results.

Our results indicate that Lahti showed improvement in the proportion of patients discharged home within 90 days. In addition, the proportion of patients institutionalized within 90 days decreased compared with the rest of Finland. This supports the finding that Finnish interprofessional stroke centres have reduced the risk of institutionalization (13). However, the results on mortality rates did not improve.

It is evident that other areas in Finland have also developed stroke care and rehabilitation during these years (14, 15). This may have stimulated the improvement of stroke rehabilitation in Finland in general and may explain some of the effects of our regional intervention in 2013. A patient organization conducted a nationwide survey of stroke rehabilitation resources in different areas in Finland (14). The efforts have still been limited and only 15–20% of stroke patients in Finland received interprofessional rehabilitation (14). A subsequent review conducted in 2016 revealed that the situation had not improved (15). Finnish wards are often described as interprofessional rehabilitation wards but in fact do not have the required resources to support proper interprofessional rehabilitation (15) Both reviews pointed out differences in rehabilitation resources between different regions (14, 15) Comparisons with different countries are challenging due to lack of data on rehabilitation services provided (17).

One important finding was that both interventions decreased the costs of first institutionalized episode compared with the rest of Finland even though interprofessional resources on the ward and among home-based rehabilitation teams were enhanced. The decreased length of the first institutionalized episode probably explains this result.

Although the study population covered nationwide hospital-treated stroke patients, a limitation is that the PERFECT performance indicators measured the stroke patient’s functioning (discharge home, institutionalization, and costs) only indirectly. Information on stroke severity (33), as well as functional ability, such as the measures of activities in daily living and stroke disability (34), were not available from Finnish administrative registers. Risk adjustment based on age and sex, and comorbidities based on the medical history of patients, may not be enough for a reliable performance comparison of diseases affecting older persons. Also, the stroke acute care has developed during the same period, which may have improved the survival rate and functioning of the stroke patients (4–6). The lack of outpatient data is also a limitation that might bias the estimation of the cost gain from the intervention.

Our study showed that the changes and improvements in stroke rehabilitation measures and in the rehabilitation pathway in Lahti and in Päijät-Häme may have impacted changes in PERFECT performance indicators (i.e., in outcomes). A similar improvement has also been reported in Lahti in the care of hip-fracture patients (21). These indicators show that Lahti achieved the level of the rest of Finland in the proportion of patients institutionalized and improved the length of first acute care, even though mortality rates were not improved. This finding suggests that the regional acute care pathway should be scrutinized more thoroughly. Further studies are needed to determine the effectiveness of stroke rehabilitation in the Päijät-Häme region in functioning and quality of life, and the cost-effectiveness of stroke rehabilitation.

In conclusion, our study indicates that intensive stroke rehabilitation and improvements of patient pathway led to faster and better return to home for patients and reduced costs for the healthcare system.

## Supplementary Material

THE EFFECTIVENESS AND COSTS OF INTENSIVE STROKE REHABILITATION AND IMPROVEMENTS IN PATIENT PATHWAY IN FINLAND: A RETROSPECTIVE BENCHMARKING CONTROLLED TRIAL

## Data Availability

Unto Häkkinen has permission to use the individual-level data used in this study and can upon reasonable request produce additional information that supports the findings of this study.
